# Diversity of honey stores and their impact on pathogenic bacteria of the honeybee, *Apis mellifera*

**DOI:** 10.1002/ece3.1252

**Published:** 2014-09-26

**Authors:** Silvio Erler, Andreas Denner, Otilia Bobiş, Eva Forsgren, Robin F A Moritz

**Affiliations:** 1 Departamentul de Apicultură şi Sericicultură, Universitatea de Ştiinţe Agricole şi Medicină VeterinarăCalea Mănăştur 3-5, 400372, Cluj-Napoca, Romania; 2 Institut für Biologie, Molekulare Ökologie, Martin-Luther-Universität Halle-WittenbergHoher Weg 4, 06099, Halle, Germany; 3 Department of Ecology, Swedish University of Agricultural SciencesP.O. Box 7044, 75007, Uppsala, Sweden; 4 Department of Zoology and Entomology, University of Pretoria0002, Pretoria, South Africa

**Keywords:** American foulbrood, antimicrobial activity, disease ecology, European foulbrood, host–parasite interaction, *Paenibacillus larvae*, self-medication

## Abstract

Honeybee colonies offer an excellent environment for microbial pathogen development. The highest virulent, colony killing, bacterial agents are *Paenibacillus larvae* causing American foulbrood (AFB), and European foulbrood (EFB) associated bacteria. Besides the innate immune defense, honeybees evolved behavioral defenses to combat infections. Foraging of antimicrobial plant compounds plays a key role for this “social immunity” behavior. Secondary plant metabolites in floral nectar are known for their antimicrobial effects. Yet, these compounds are highly plant specific, and the effects on bee health will depend on the floral origin of the honey produced. As worker bees not only feed themselves, but also the larvae and other colony members, honey is a prime candidate acting as self-medication agent in honeybee colonies to prevent or decrease infections. Here, we test eight AFB and EFB bacterial strains and the growth inhibitory activity of three honey types. Using a high-throughput cell growth assay, we show that all honeys have high growth inhibitory activity and the two monofloral honeys appeared to be strain specific. The specificity of the monofloral honeys and the strong antimicrobial potential of the polyfloral honey suggest that the diversity of honeys in the honey stores of a colony may be highly adaptive for its “social immunity” against the highly diverse suite of pathogens encountered in nature. This ecological diversity may therefore operate similar to the well-known effects of host genetic variance in the arms race between host and parasite.

## Introduction

Colonies of highly eusocial insects are particularly attractive for various suites of microbial pathogens (Schmid-Hempel 1998). Nest homeostasis, that is, constant temperature, humidity and respiratory gases, as well as rich food stores and vast amounts of brood create excellent growth conditions for microorganisms. Furthermore, the large number of closely related individuals in tight proximity with high interaction frequencies promotes pathogen transmission (Schmid-Hempel 1998). In particular, colonies of the cave breeding honeybee (*Apis mellifera)* have extremely well controlled intracolonial homeostasis (35°C and 60% relative humidity) providing ideal conditions for growth of bacterial pathogens. So, it is not surprising that specialized bacterial pathogens such as *Paenibacillus larvae*, the infectious agent of American foulbrood (AFB) can easily infect brood, multiply, and successfully spread through the entire colony. Young larvae become infected with bacterial spores from larval food (White 1906). When reaching the larval midgut, the spores germinate and proliferate. Eventually, the honeybee larva dies from bacteramia and lysis of its organs before pupation (Davidson 1973; Genersch et al. 2005). Billions of new spores produced in the larval remains are spread by the workers cleaning the cells and feeding other larvae, eventually leading to colony depletion (Fries et al. 2006; Lindström et al. 2008). As also beekeeping equipment and bee products may be contaminated with spores, the disease can easily spread to neighboring colonies (Shimanuki 1983). AFB is a major problem in apiculture, particularly because *P. larvae* has become increasingly resistant to common antibiotics like oxytetracycline, still used in apiculture in some countries (Kochansky et al. 2001; Evans 2003). Today, *P. larvae* has been classified into four genotypes ERIC I–IV, which show specific differences not only in phenotype, but also in virulence (Genersch et al. 2006).

Although AFB is arguably the most virulent bacterial threat to honeybee colonies also European foulbrood (EFB), a bacterial gut infection, may lead to larval death before pupation and can cause occasional colony losses (Tarr 1936; Bailey 1983). Highly infectious larval remains are cleaned out from the cells by nurse bees, which may spread the pathogens to other nest members (Forsgren 2010). In addition to the major bacterial pathogen *Melissococcus plutonius*, secondary bacterial invaders often co-occur with EFB disease, including *Enterococcus faecalis*, *Paenibacillus alvei*, *Brevibacillus laterosporus*, *Bacillus pumilus*, and *Achromobacter euridice* (Forsgren 2010).

The release of the genome sequence of *A. mellifera* (Honeybee Genome Sequencing Consortium 2006) revealed that honeybees have a reduced set of immune genes compared to other insect species (Evans et al. 2006). This deficiency can partially be compensated by “social immunity” (Cremer et al. 2007) resulting from highly adaptive behavior of workers towards infected colony members. Already Rothenbuhler (1964) showed that workers are able to detect and remove infected larvae or parasitized pupae. This hygienic behavior eventually results in resistance of colonies to *P. larvae* (Rothenbuhler and Thompson 1956; Rothenbuhler 1964; Spivak and Gilliam 1998; Wilson-Rich et al. 2009). Simone et al. (2009) showed that collecting antimicrobial propolis (plant resins collected by honeybees) may further contribute to the “social immunity” at colony level as the expression of immune-related genes decreased after exposing workers to propolis (Simone et al. 2009; Simone and Spivak 2010).

It is long known that honey has a potent antimicrobial activity, and it has been used since ancient times by humans for treatment of wound infections caused by pathogens (Aristotle 384-322 BC; Bogdanov et al. 2008). In particular, the high sugar concentrations (80%) in honey, mainly glucose and fructose, result in strong antimicrobial properties due to the extreme osmotic stress for pathogens (Molan 1992). However, the antimicrobial quality of honey is not just due to the sugar concentration alone. During honey production, glucose oxidase is added by honeybees to nectar. This enzyme converts glucose to gluconic acid and hydrogen peroxide (H_2_O_2_), which is known to have potent antimicrobial activity (White et al. 1963). Moreover, secreted antimicrobial proteins from unique lactic acid producing microbiota found in the honey stomach may also contribute to the differences in antimicrobial properties of honey (Olofsson and Vásquez 2008; Butler et al. 2013). Finally, nectar contains many secondary plant metabolites including various aromatic acids and diverse phenols (polyphenols and flavonoids) with high antimicrobial activity (Molan 1992; Bogdanov 1997; Cowan 1999; González-Teuber and Heil 2009). These plant derived honey compounds are not only highly plant specific, but also depend on seasonal and environmental factors as well as processing and storage by bees (Kaškonien≐ and Venskutonis 2010). Under natural conditions, when the beekeeper does not extract the honey from the hive, the honey stores of a colony will therefore contain a variety of honeys from many different plant sources with variable composition of secondary plant metabolites and also variance in antimicrobial competence. Once stored inside the hive, the different honeys are available independent of the foraging season. Hence, different honeys can potentially be chosen by the worker bees not only to satisfy their carbohydrate needs for food, but also for their antimicrobial activity (Gherman et al. 2014).

Given this variance in antimicrobial compounds among different honeys, they may well have specific efficacies against various bacterial pathogens. It would be highly adaptive if honeybees could take advantage of this variability in the honey store using the different honeys to fight various pathogens. The use of this potential for an efficient selective self-medication might considerably increase the “social immunity” of the colony. Indeed, honeybees have been shown to selectively choose among several honey types depending on the health status (Gherman et al. 2014). Parasite infected workers preferred those honeys, which had a higher potential to reduce the infection. Although these results were experimentally achieved under laboratory conditions, self-medication by selectively using the honey stores cannot be excluded as an important mechanism for “social immunity”. We here study in vitro antibiotic effects of honeys from different floral sources on the growth of various bacteria involved in AFB and EFB, and whether the specificity and diversity of various honeys in the colony can contribute to overall colony immunity.

## Material and Methods

### Bacterial strains and cultivation

All bacteria used in this study were Gram-positive bacteria and provided by BCCM/LMG Bacteria Collection (Ghent University, Ghent, Belgium). The bacterial strains *Paenibacillus larvae* LMG 9820 (ERIC I), *P. larvae* LMG 16252 (ERIC III), and *P. larvae* LMG15974 (ERIC IV), the cause of American foulbrood (AFB), were used as model organisms. For the study of European foulbrood (EFB), *Melissococcus plutonius* LMG 20360, *Enterococcus faecalis* LMG 7937, *Paenibacillus alvei* LMG 13253, *Brevibacillus laterosporus* LMG 16000, and *Bacillus pumilus* (SLU 119-12), isolated from diseased brood with EFB symptoms, a so far unknown bacterial species associated with EFB, were used. Vacuum-sealed ampoules with freeze-dried bacterial cultures were opened, and 0.5 mL of appropriate standard cultivation broth (according to the BCCM/LMG Bacteria Collection instructions) were added using a sterile Pasteur pipette. The content was mixed and transferred to solid and liquid media. All tested bacteria were grown under aerobic conditions and *Melissococcus plutonius* in a micro-aerophilic milieu (Forsgren et al. 2013) at 37°C. Bacterial suspensions were stored at −80°C with 15% sterile glycerol for subsequent use. Furthermore, bacteria species were verified using molecular tools (see Appendix S1 in Supporting Information).

### Bacterial growth assay

Following two additional subsequent cultivation steps upon initial cultivation in liquid media, as recommended by the supplier, bacterial strains were cultivated at 37°C under continuous medium speed shaking and sterile conditions in 96-well microtiter plates with a start OD_600_ (optical density) of 0.001 in 200 μL of the appropriate medium. To determine the inhibitory growth effect of different honeys on AFB and EFB specific bacteria, the following three honeys (provided by a single migratory beekeeper) were added to a final concentration of 5, 10, 25, or 50% to the broth in wells containing the honeybee pathogens: black locust (*Robinia pseudoacacia*; Valea lui Mihai, Bihor, Romania), sunflower (*Helianthus annuus*; Ciucurova, Tulcea, Romania), and a polyfloral honey (Transylvanian plain, Romania). Honeys were selected based on the specific flowering time (black locust: May–June; sunflower: July–October) avoiding overlap of nectar availability for the monofloral honeys. All honeys were proven to be free of antibiotic contaminants (e.g., tetracycline and oxytetracycline), for details see Gherman et al. (2014).

To control for the inhibitory effect of the osmosis, we added a sugar control solution (0.42 g/mL fructose, 0.32 g/mL glucose). The sugar control used in the experiment had the same concentrations as honey. Cultivation OD was continuously measured at 600 nm for 15 h every 15 min, using a fully automated plate reader (Synergy 2 Multi-Mode Microplate Reader, BioTek, Winooski, VT) and the Gen5 software (BioTek Instruments). Every treatment was measured at least five times for biological replicates.

We used the slope of the growth curve during the log phase using at least four consecutive data points using the analysis program of Ramakers et al. (2003) and standard spreadsheet software. We determined the inhibition (*I*) of bacterial growth by compounds other than sugar in relation to the inhibition of the sugar control as follows:


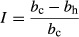
(1)

with

*I* = inhibition in relation to sugar control

*b*_h_ = slope of log phase growth honey

*b*_c_ = slope of log phase growth sugar control

For statistical analysis, we used a general linearized model (gamma-distribution and log link-function), with square root (growth inhibition + 0.5) transformed data to assess the effects of honey type or bacteria strain or the interaction of both using STATISTICA 8.0 (StatSoft, Tulsa, OK).

### Physicochemical analysis of honey

Honey authenticity was determined by standard melissopalynological analysis (Louveaux et al. 1978), counting the specific pollen grains from the sediment. Pollen spectrum was evaluated using a Nikon Eclipse 50i (Japan) optical microscope at 40 × magnification (40/0.65). Specific pollen types were identified using reference preparations and identification books (Sawyer 1981). The melissopalynological analysis of the polyfloral honey showed a mixture of maize (*Zea mays*), common dandelion (*Taraxacum officinale*), cornflower (*Centaurea cyanus*), spiny plumeless thistle (*Carduus acanthoides*), wild thyme (*Thymus serpyllum*), common hawthorn (*Crataegus monogyna*), meadowsweet (*Filipendula ulmaria*), common sainfoin (*Onobrychis viciifolia*), rapeseed (*Brassica napus*) and sunflower (*Helianthus annuus*) pollen, and honeydew elements of about 10% from the total pollen number. All identified pollen had a concentration below 5% of the total pollen sample.

Honey quality was assessed using the methods proposed by the International Honey Commission and as described in Dezmirean et al. (2012). This included the analyses of acidity, sugar composition, α-amylase, hydroxymethylfurfural (HMF), antioxidant activity (DPPH (2,2-diphenyl-1-picrylhydrazyl) assay), total polyphenol, and flavonoid content. H_2_O_2_ concentration was quantitatively determined using a colorimetric assay modified from Kwakman et al. (2010). Reagent solution (67 μL), consisting of 50 μg/mL *O*-dianisidine (Sigma-Aldrich, St. Luis, MO) and 20 μg/mL horseradish peroxidase (Sigma-Aldrich) in 10 mmol/L phosphate buffer (pH 6.5), was mixed with 20 μL diluted honey samples (0.1 g/mL). *O*-dianisidine solution was freshly prepared from a 1 mg/mL stock in demineralized water and peroxidase solution from a 10 mg/mL stock in 10 mmol/L phosphate buffer (pH 6.5). The reactions were stopped with 60 μL 6 mol/L H_2_SO_4_ after 5 min incubation at room temperature. The final absorbance was measured in five replicates at 540 nm with the Synergy 2 Multi-Mode Microplate Reader and Gen5 software. Five replicates of H_2_O_2_ standards ranging from 0.5–75 μmol/mL were made and 20 μL of each standard were added to each plate. H_2_O_2_ concentration of each honey was calculated using the calibration curve (Fig. S1).

## Results

### Verification of bacterial strains and honeys

All PCR products of the 16S rRNA gene had the expected sizes. DNA nucleotide sequences and BLAST results (using NCBI nucleotide BLAST) confirmed the identity of the tested strains.

The results of physicochemical analyses for all tested honeys (pH, water content, acidity, sugar content, hydroxymethylfurfural, and diastase activity) fell within the limits set by the European Commission (EEC, 110/2001) (Table S1). However, factors known to be related to the antimicrobial activity of honey showed a high variance among the tested samples. Antioxidant activity ranged from 15.7% (black locust) to 28.9% (sunflower) inhibition of the DPPH radical. The total phenolic content was between 34.8 mg/100 g (black locust) and 84.7 mg/100 g gallic acid equivalents (polyfloral). The total flavonoid content expressed in quercetin equivalents (QE) varied between 10.2 QE in black locust and 20.0 QE in polyfloral honey (Table S1). Hydrogen peroxide had the highest concentration in polyfloral honey (3.81 μg/mL H_2_O_2_) but was below the detection limits in sunflower honey (Table S1).

### Effect of sugar

**Figure 1:**
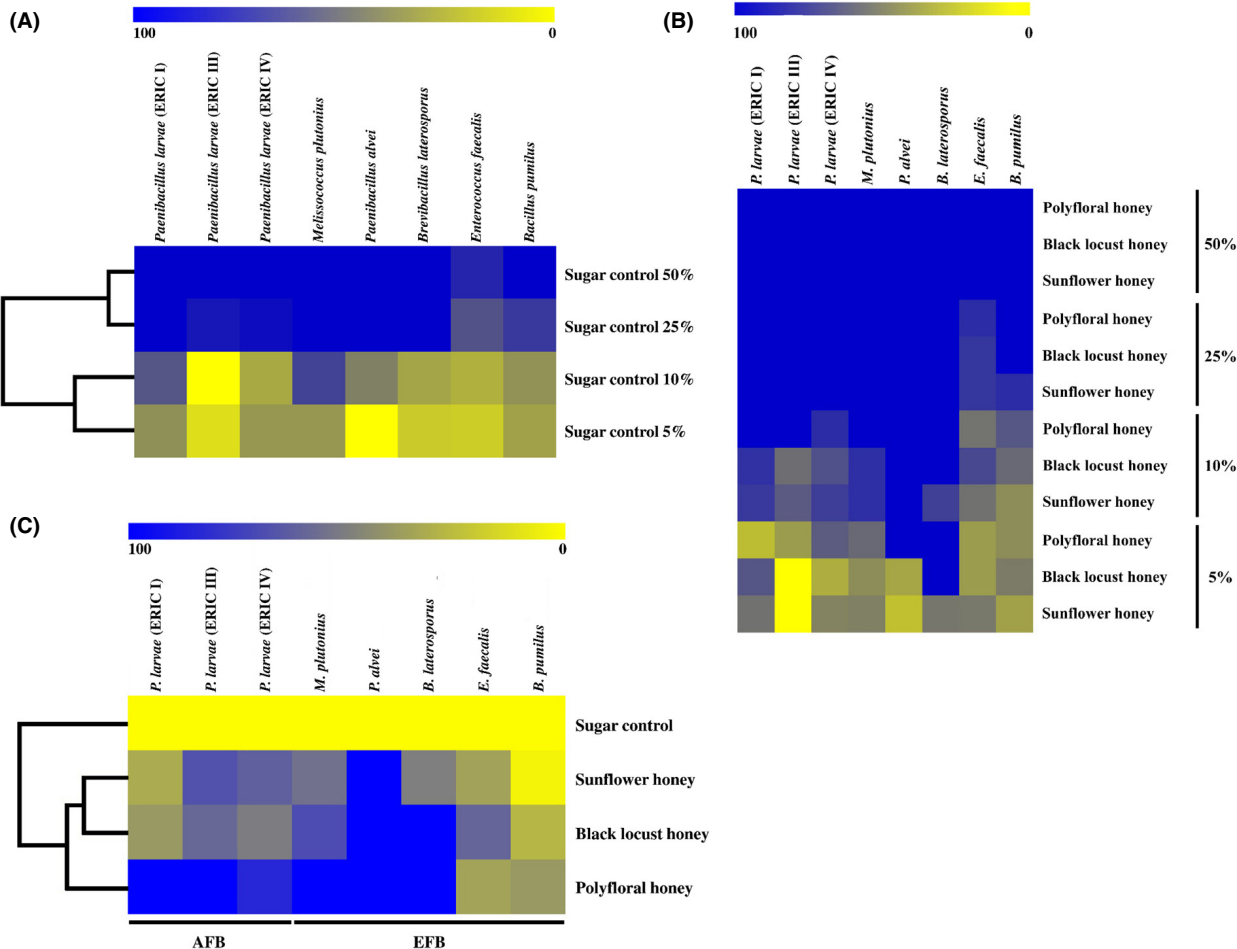
Growth inhibitory effect of sugar and honey (polyfloral, sunflower, and black locust) on AFB causing, EFB causing and associated bacterial strains. (A) Sugar normalized to their untreated positive control; (B) honey samples normalized to their untreated positive control; and (C) only 10% honey samples, normalized to the bacteria growth observed when treated with 10% sugar.

Except for *E. faecalis* (86% inhibition compared to the positive control) all other bacteria were completely inhibited by the 50% sugar control. *P. larvae* ERIC III, ERIC IV, *E. faecalis*, and *B. pumilus* were partially inhibited by the 25% sugar solution (Fig.1A, Table S2). *E. faecalis* and *B. pumilus* were inhibited with more than 65%, but the two AFB strains still more than 90%. Even in the media containing 5 and 10% sugar most bacterial strains showed growth inhibition. However, at these sugar concentrations inhibition was highly variable ranging between −4% growth inhibition for *P. larvae* (ERIC III) and 74% for *M. plutonius* (Fig.1A, Table S2).

### Effect of honey

In general, the growth inhibitory effect increased with increasing honey concentration for all bacterial strains (Fig.1B, Table S2). All AFB bacteria strains, EFB-associated strains (*E. faecalis*, *B. pumilus*) and *M. plutonius* were able to grow on media containing 5% honey (Fig.1B, Table S2), but showed up to 67% growth inhibition compared to their respective positive controls.

The highest variance for growth inhibition among the screened bacteria was observed at a concentration of 10% honey (Fig.1B, Table S2). At this concentration polyfloral honey inhibited bacterial growth completely for all AFB and EFB specific strains except for the *P. larvae* strain ERIC IV (83% inhibition). Sunflower and black locust honey were not able to inhibit any AFB or *M. plutonius* strain completely, but sunflower honey reduced the bacterial growth of *P. larvae* ERIC III, and ERIC IV more effectively, and increased the growth of *P. larvae* ERIC I compared to black locust honey. *M. plutonius* growth showed no difference with 10% sunflower and black locust honey.

Most bacteria showed complete growth inhibition in media with 50 and 25% honey. Only *E. faecalis* (<85% growth inhibition on 25% honey) and *B. pumilus* were able to grow on 25% sunflower honey (Fig.1B, Table S2).

*Brevibacillus laterosporus* was completely inhibited on polyfloral and black locust honey at all concentrations and suffered strong inhibitory effects (>50%) in media with 5 and 10% sunflower honey. The growth of *P. alvei* was completely inhibited in all concentrations of polyfloral honey.

### Inhibitory effect of compounds other than sugar

We also compared the inhibitory effect (*I*) of the three honeys with those of the sugar controls to reveal antibiotic effects not due to the osmotic effect alone. Here, we focused on the inhibition at the 10% concentration levels (Fig.1B) because of the high pathogen specific variance among the inhibitory effects of the different honeys. Using a generalized linear model, honey type, bacterial strain, as well as the interaction between both, all had a highly significant impact (*P* < 0.0001) on the inhibition of bacterial growth. The highly significant interaction component of honey x bacteria shows that specific honey types are inhibiting specific pathogenic bacteria both for AFB (*W* = 89.618, df = 4, *P* < 0.0001) and EFB (*W* = 377.151, df = 8, *P* < 0.0001).

Figure1C shows the inhibitory effect of 10% honey treatment on AFB and EFB bacterial strains. Polyfloral honey almost completely inhibited bacterial growth of all AFB and the EFB-associated strains (>84%). Only *E. faecalis* and *B. pumilus* were less inhibited <40%. Black locust honey completely inhibited *P. alvei* and *B. laterosporus*. The same effect was observed for sunflower honey and *P. alvei*.

The inhibition of black locust and sunflower honey appeared to be strain specific. On the one hand, black locust honey inhibited EFB-specific and EFB-associated bacteria much more effectively than sunflower honey (MWU-test, *U* = 3024, *P* < 0.0001). On the other hand, sunflower honey inhibited the AFB strains more strongly than black locust honey (MW *U*-test, *U* = 713.5, *P* = 0.0013) (Fig.1C).

In a final multivariate analysis, we tested if any of the analyzed physicochemical parameters of the honeys might explain the observed specific pattern of bacterial growth inhibition. However, neither a principal component nor multiple regression analysis revealed any significant interaction (Bonferroni adjusted *P*-value > 0.008).

## Discussion

### Effect of sugar

Sugar is a natural source of energy for both the honeybee and the tested bacteria, but it can also build up high osmotic pressure that inhibits bacterial cell growth. However, our results clearly confirm that the sugar concentration alone is insufficient to explain all antibiotic effects of honey. Particularly at low concentrations, honey showed a substantially stronger antimicrobial effect than sugar alone. Although the sugar concentration in the stored honey is high (≥80%), the honey fed by nurse bees to the larvae is highly diluted. Already von Planta (1889) and Asencot and Lensky (1988) showed that total sugar concentrations in the larval food (worker jelly), depending on the developmental stage, range between 5% and 13% for worker larvae, which is well below the critical sugar concentrations that fully inhibit bacterial growth. That means worker larvae are fed with comparable sugar concentrations as used in our study. Honey diluted to 5 and 10% has an equal amount of sugar (comparing glucose, fructose, and saccharose) as the worker jelly, at least in the same order of magnitude (von Planta 1889; Asencot and Lensky 1988).

### Effect of monofloral honey on AFB and EFB

The most striking effects were obtained at 10% honey concentrations, where the difference between the tested honey and the sugar solutions were highest. The monofloral honeys differed significantly in their antimicrobial activity against specific AFB and EFB associated bacterial strains. Whereas black locust honey showed a strong and highly significant inhibitory effect on AFB, sunflower honey inhibited EFB most strongly. However, none of the known antimicrobial substances in honey (Kwakman et al. 2010) showed a significant correlation with the growth inhibitory pattern against AFB and EFB associated bacterial strains. Hence compounds other than those included in the standard physicochemical analysis or an additive effect of tested and nontested compounds are likely to add to the antimicrobial effects. Indeed many secondary plant metabolites with known antimicrobial potential have been found in honey (Adler 2000). These compounds have been shown to be highly plant specific and include radical scavenging activity, polyphenols and flavonoids that interfere with pathogen growth (Cushnie and Lamb 2005). The strength of antimicrobial effects can also depend on the interaction among different flavonoids (Mihai et al. 2012). Further, honeybee specific lactic acid bacteria have been shown to play an important role in producing antimicrobial substances in honey (Butler et al. 2013). The composition of the lactic acid bacteria microflora varies depending on floral sources, thus the substances they produce (Olofsson and Vásquez 2008).

### Effect of polyfloral honey

Polyfloral honey almost completely inhibited bacterial growth of all AFB and EFB strains at honey concentrations ≥10%. At every tested honey concentration, the strongest growth inhibition was observed. Whereas none of the standard compounds correlated with this increased antimicrobial potential, the polyfloral honey did have about the double polyphenol concentration compared to the monofloral honeys. This might explain the enhanced antimicrobial activity; however, it might also be due to a combination of other compounds similar to those (yet undetected) that facilitated the specific effects on the various bacteria in the monofloral honeys. Beside different plant species identified in melissopalynological analysis of polyfloral honey, 10% of the sediment compounds were honeydew elements that may influence the antimicrobial activity of the honey (Bogdanov 1997). Although the polyfloral honey showed a high antimicrobial potential, one must, however, acknowledge that it can also harbor the highest microbial diversity (bacteria associated with the bees’ environment) (Sinacori et al. 2014).

### Relevance of diverse honey stores for colony health

During the season, foragers sequentially collect a most diverse set of floral nectar, which is stored in the honey combs. The colony's food source selectivity in terms of nectar, as selective decision behavior by foraging bees, is a process of natural selection among alternative nectar sources including effectiveness and communication (Seeley et al. 1991). Hence, the workers may also choose from a complex mix of different honeys because honey stores will overlap with seasonally changing flower availability. Singaravelan et al. (2005) showed that low concentrations of secondary compounds elicit a significant feeding preference, confirming the mechanism of selectively choosing between specific nectar resources regardless of availability. This qualitative variance in the honey stores of the colony may be of considerable importance for colony health whenever it is exposed to various pathogenic bacteria. As honey is the central nutrient for developing larvae, the diversity in the honey stores may serve as a richly stocked natural “in-hive pharmacy” against a broad variety of brood diseases. During the first 2 days after hatching from the egg, the larval diet mainly consists of components secreted from the hypopharyngeal food glands of nurse bees, presumably mixed with honey. However, beginning with the third day honey and pollen is added to the diet and directly fed to the worker larvae (Winston 1987). Thus, the nurse bees are in the central position of the intracolonial food web, and might provide a mechanism to promote the colony's health status by selectively feeding specific honeys in response to specific infections. Indeed, nurse bees have been shown to adaptively choose honey based on their own health status (Gherman et al. 2014). Also other studies on honeybees (Simone-Finstrom and Spivak 2012), but also wood ants (Christe et al. 2003; Chapuisat et al. 2007) have shown that workers collect more plant derived products as prophylactic use to protect the colony. Furthermore, honeybees have been shown to selectively forage among specific resins, even discriminating closely related resinous plants (Wilson et al. 2013). The authors concluded that honeybees can make discrete choices among resinous plant species, further confirming selective preference among specific health promoting resources regardless of their availability.

If variable honey stores facilitate colony health, this would not only be an important evolutionary achievement of honeybee colonies, it would also have profound consequences for beekeeping practices. Apiculturists might take advantage of specific honey flows to protect their colonies against specific diseases. In addition, beekeepers should be aware that the exclusive production of monofloral honeys may have negative consequences for colony health. Also, the feeding of sugar as a food source over winter may enhance the propensity of the colony to be infected by pathogens. In conclusion, floral biodiversity providing the nectar source for the colony will have direct implication for colony health with similar importance as the genetic diversity of the honeybee.

## Supporting Information

**Appendix S1**. Bacteria species verification. **Figure S1**. Calibration curve to determine hydrogen peroxide content. **Table S1**. Physicochemical analysis of different honeys. **Table S2**. Maximal slopes from growth curves (*b* = mean × 10^−3^ ± SE × 10^−3^) of bacterial cultivation with sugar and honey treatment, and growth inhibition (*I*) in % of the positive control.
