# Supplementary material for: Diversity of honey stores and their impact on pathogenic bacteria of the honeybee, Apis mellifera
Source: Ecol Evol. 2014 Sep 26;4(20):3960–7. doi: 10.1002/ece3.1252 (PMC4242578; doi:10.1002/ece3.1252)
Supplement: Supplementary file 1 [file ece30004-3960-SD1.pdf]

Diversity of honey stores and their impact on pathogenic bacteria of the honeybee, *Apis mellifera*

Supporting Information

Appendix S1: Bacteria species verification

DNA was isolated from pathogens using the DNeasy Blood & Tissue Kit (Qiagen, Hilden, Germany) protocol for gram-positive and -negative bacteria, from bacterial cultures with an OD_{600} higher than 1. DNA was eluted in 100 µL sterile water and stored at -20 °C until further processing.

To verify whether the bacterial strains used were not contaminated after several sub-culturing steps, amplification of bacterial DNA with universal bacteria 16sRNA primer (Simone et al., 2009) was done using PCR Master Mix (Thermo Fisher Scientific, Bremen, Germany), 0.2 µM of each primer and 1 µl diluted bacterial DNA (1:100); and the following PCR protocol: 95 °C for 3 min; 35 cycles with 95 °C for 1 min, 57 °C for 30 sec, 72 °C for 1 min; and final elongation at 72 °C for 5 min. PCR product size was verified by automated capillary electrophoresis (QIAxcel system) using the High Resolution Cartridge (Qiagen, Hilden, Germany). PCR products were purified using Sure Clean (Bioline, Luckenwalde, Germany) and sent to Macrogen (Amsterdam, Netherlands) for sequencing.

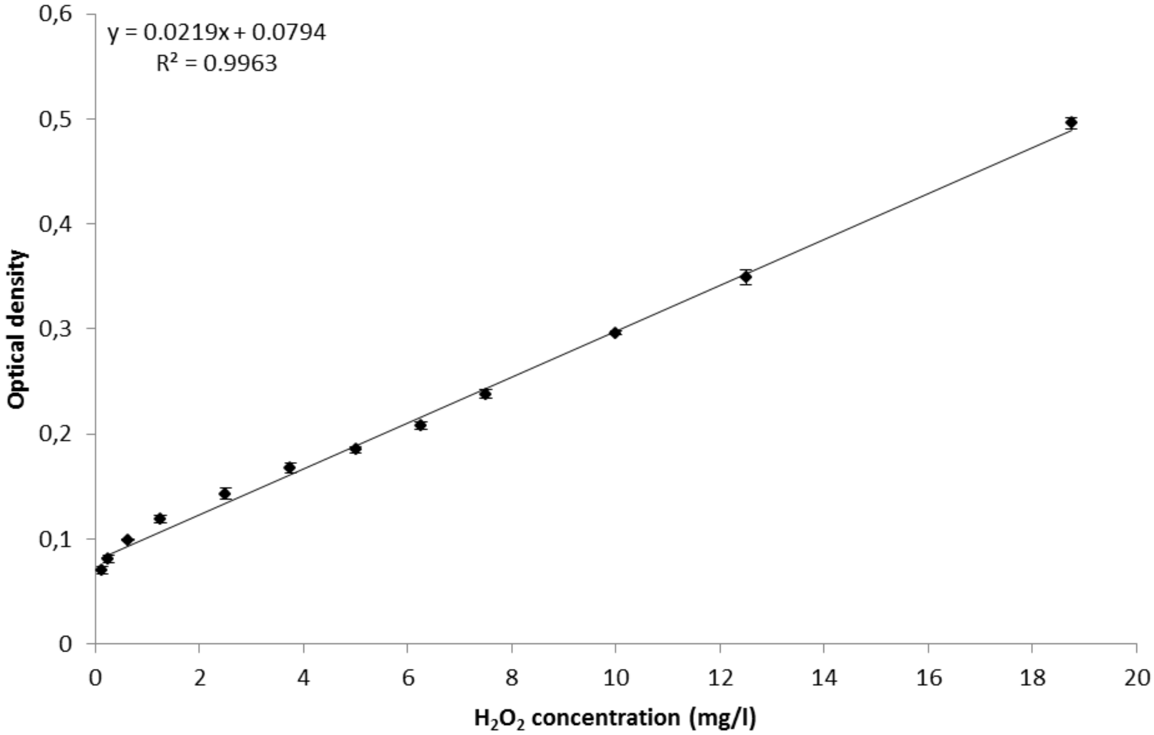

Fig. S1 Calibration curve to determine hydrogen peroxide content. Five replicates were used for each data point (Mean ± SE).

Table S1 Physicochemical analysis of different honeys.

Analysed parameter	Honey type		
	Polyfloral	Sunflower	Black locust
pH	4.0	3.8	4.0
Water content (%)	16.8	18.1	16.0
Free acidity (meq/kg)	17.0	25.7	6.5
Lactonic acidity (meq/kg)	13.0	21.9	5.9
Total acidity (meq/kg)	30.0	47.6	12.4
Glucose (%)	39.3	38.4	28.0
Fructose (%)	38.9	39.0	44.0
Sucrose (%)	0.3	0.1	0.8
Turanose (%)	1.2	1.1	2.5
Maltose (%)	2.7	1.5	4.0
Isomaltose (%)	0.3	0.2	0.5
Fructose/glucose	1.0	1.0	1.6
Total sugar (%)	82.8	80.3	79.8
Diastase activity (Schade units/g)	49.3	22.6	28.5
Hydroxymethylfurfural (mg/kg)	0.4	8.5	2.7
Antioxidant activity (DPPH assay) (% inhibition)	17.4	28.9	15.7
Total polyphenols (gallic acid equivalent) (mg/100 g)	84.7	41.5	34.8
Total flavonoids (quercetin equivalent) (mg/100 g)	20.0	19.5	10.2
H_2O_2 (mg/l)	3.81	n.d.	3.62

meq, milliequivalent; n.d., not detectable

Table S2 Maximal slopes from growth curves (b = mean $\times10^{-3}$ ± SE $\times10^{-3}$) of bacterial cultivation with sugar and honey treatment, and growth inhibition (I) in % of the positive control. Slopes are shown as absolute values.

		***P. larvae* ERIC I**		***P. larvae* ERIC III**		***P. larvae* ERIC IV**		***M. plutonius***		***P. alvei***		***B. laterosporus***		***E. faecalis***		***B. pumilus***	
		b	*I*	*b*	*I*	*b*	*I*	*b*	*I*	*b*	*I*	*b*	*I*	*b*	*I*	*b*	*I*
**Positive control**		27 ± 4	0	23 ± 0.5	0	59 ± 7	0	22 ± 4	0	20 ± 0.2	0	28 ± 6	0	127 ± 5	0	88 ± 9	0
**Sugar**	50%	0	100	0	100	0	100	0	100	0	100	0	100	18 ± 2	86	0	100
	25%	0	100	2 ± 0.6	91	3 ± 0.7	95	0	100	0	100	0	100	41 ± 2	68	19 ± 5	78
	10%	9 ± 2	67	24 ± 4	-4	39 ± 2	34	9 ± 4	74	10 ± 0.6	50	18 ± 4	36	88 ± 1	31	50 ± 3	43
	5%	15 ± 1	44	20 ± 2	13	35 ± 2	41	13 ± 6	41	20 ± 0.3	0	22 ± 1	21	102 ± 7	20	63 ± 4	37
**Polyfloral honey**	50%	0	100	0	100	0	100	0	100	0	100	0	100	0	100	0	100
	25%	0	100	0	100	0	100	0	100	0	100	0	100	22 ± 1	83	0	100
	10%	0	100	0	100	10 ± 4	83	0	100	0	100	0	100	57 ± 2	55	30 ± 2	66
	5%	20 ± 5	26	14 ± 1	39	21 ± 4	64	9 ± 4	59	0	100	0	100	77 ± 5	39	48 ± 4	45
**Black locust honey**	50%	0	100	0	100	0	100	0	100	0	100	0	100	0	100	0	100
	25%	0	100	0	100	0	100	0	100	0	100	0	100	27 ± 0.3	79	0	100
	10%	5 ± 5	81	10 ± 0.6	57	18 ± 1	69	3 ± 8	82	0	100	0	100	35 ± 0.2	72	36 ± 1	59
	5%	9 ± 9	67	23 ± 0.5	0	40 ± 2	32	12 ± 6	45	13 ± 1	35	0	100	77 ± 1	39	42 ± 4	52
**Sunflower honey**	50%	0	100	0	100	0	100	0	100	0	100	0	100	0	100	0	100
	25%	0	100	0	100	0	100	0	100	0	100	0	100	27 ± 0.6	79	15 ± 0.5	83
	10%	6 ± 1	78	8 ± 0.3	65	14 ± 0.3	76	4 ± 8	82	0	100	7 ± 3	75	56 ± 1	56	48 ± 6	45
	5%	12 ± 0.8	56	23 ± 2	0	30 ± 3	49	11 ± 1	50	15 ± 0.4	25	13 ± 2	54	60 ± 4	53	55 ± 1	37

b, slope $\times10^{-3}$ of the linear growth curve

Reference

Simone, M., Evans, J.D. & Spivak, M. (2009). Resin collection and social immunity in honey bees. *Evolution*, 63, 3016-3022
